# Hepatitis B Virus and Tuberculosis Are Associated with Increased Noncommunicable Disease Risk among Treatment-Naïve People with HIV: Opportunities for Prevention, Early Detection and Management of Comorbidities in Sierra Leone

**DOI:** 10.3390/jcm11123466

**Published:** 2022-06-16

**Authors:** George A. Yendewa, Sulaiman Lakoh, Darlinda F. Jiba, Sahr A. Yendewa, Umu Barrie, Gibrilla F. Deen, Mohamed Samai, Jeffrey M. Jacobson, Foday Sahr, Robert A. Salata

**Affiliations:** 1 Department of Medicine, Case Western Reserve University School of Medicine, Cleveland, OH 44106, USA; jxj573@case.edu (J.M.J.); robert.salata@uhhospitals.org (R.A.S.); 2 Division of Infectious Diseases and HIV Medicine, University Hospitals Cleveland Medical Center, Cleveland, OH 44106, USA; 3 Johns Hopkins Bloomberg School of Public Health, Baltimore, MD 21205, USA; 4 Department of Medicine, College of Medicine and Allied Health Sciences, University of Sierra Leone, Freetown, Sierra Leone; lakoh2009@gmail.com (S.L.); gibrilladeen1960@yahoo.com (G.F.D.); dhmsamai@yahoo.co.uk (M.S.); fsahr65@gmail.com (F.S.); 5 Ministry of Health and Sanitation, Freetown, Sierra Leone; darlindajiba.dj@gmail.com (D.F.J.); syendewa@gmail.com (S.A.Y.); 6 Infectious Disease Research Network, Freetown, Sierra Leone; barrieumu1993@gmail.com

**Keywords:** HIV, HBV, tuberculosis, noncommunicable diseases, Sierra Leone

## Abstract

Noncommunicable diseases (NCDs) are a growing public health concern in low- and middle-income countries and disproportionately affect people living with HIV (PWH). Hepatitis B virus (HBV) and tuberculosis (TB) coinfection are presumed risk factors in endemic settings; however, supporting evidence is conflicting. We analyzed baseline data of newly diagnosed PWH prospectively enrolled in the Sierra Leone HIV Cohort Study in Freetown, Sierra Leone, from March to September 2021. Logistic regression was used to identify associations between NCDs, HBV and TB. A total of 275 PWH aged ≥18 years were studied (55% female, median age 33 years, median CD4 307 cells/mm^3^, 15.3% HIV/HBV, 8.7% HIV/TB). NCDs were bimodally distributed, with 1 in 4 PWH clustered around liver disease (fibrosis/cirrhosis), diabetes/prediabetes and obesity/preobesity, while 1 in 8 had renal impairment or hypertension (HTN). Overall, 41.5% had ≥1 NCD, while 17.5% were multimorbid (≥2 NCDs). After adjusting for age, sex, sociodemographic factors and CD4 count, liver fibrosis/cirrhosis was strongly associated with HBV (aOR 8.80, 95% CI [2.46–31.45]; *p* < 0.001) and diabetes/prediabetes (aOR 9.89, 95% CI [1.14–85.67]; *p* < 0.037). TB independently predicted diabetes/prediabetes (aOR 7.34, 95% CI [1.87–28.74]; *p* < 0.004), while renal impairment was associated with proteinuria (aOR 9.34, 95% CI [2.01–43.78]; *p* < 0.004) and HTN (aOR 6.00, 95% CI [1.10–35.39]; *p* < 0.049). Our findings warrant the implementation of NCD-aware HIV programs for the prevention, early detection and management of comorbidities.

## 1. Introduction

Globally, an estimated 38 million people were reported to be infected with the human immunodeficiency virus (HIV) in 2021, with two-thirds residing in sub-Saharan Africa (SSA) [1]. Simultaneously, SSA accounts for 18% and 29% of the global burden of hepatitis B virus (HBV) and tuberculosis (TB), respectively [2,3]. Due to the overlap of all three epidemics in SSA, coinfections are common and serve as important determinants of morbidity and mortality in this region [2,3]. HIV/HBV coinfected individuals experience high rates of immune reconstitution upon initiating antiretroviral therapy (ART) [4] and an accelerated pace of progression to acquired immunodeficiency syndrome (AIDS) [5], liver cirrhosis and hepatocellular carcinoma [6]. Additionally, HIV infection is a well-recognized risk factor for the activation of latent TB infection, increasing the risk of progression to active TB disease 18-fold [3].

The widespread availability of ART, in addition to improved screening and management strategies for coinfections have substantially reduced HIV-related morbidity and prolonged life expectancy [1]. Paradoxically, this epidemiologic transition has been accompanied by an increase in the burden of noncommunicable diseases (NCDs) among people living with HIV (PWH) [7]. NCDs account for an estimated 41 million deaths annually, with 77% of these occurring in low- and middle-income countries (LMICs) [8]. According to a recent meta-analysis by Patel et al. [9], the most common NCDs among PWH in LMICs include cardiovascular disease (CVD), cervical cancer, depression and diabetes (DM). Studies from high-income countries have identified HIV-induced inflammation [10], ART-related toxicities [11,12] and a high prevalence of lifestyle-associated risk factors such as excessive alcohol use, tobacco smoking, unhealthy dietary habits and reduced physical activity as contributing to NCDs in an aging cohort of PWH [10,11,12]. However, there is limited research on the prevalence, correlates and pathogenesis of NCDs among PWH in LMICs. While a heightened proinflammatory state and immune dysregulation induced by coinfections such as viral hepatitis [13] and TB [14] have been suggested as possible risk factors in endemic settings, their relative contributions to the pathogenesis of NCDs have not been quantified.

Sierra Leone is a West African country with high HBV endemicity (estimated population prevalence of 8–10%) [15,16,17] and one of 30 high TB burden countries [18] in the setting of a generalized HIV epidemic [19]. Although the HIV epidemic in Sierra Leone has not been fully characterized, preliminary studies have revealed that the majority of PWH (up to 75%) present with late-stage disease (defined as CD4 < 350 cells/mm^3^) [20]. Other prominent features of the HIV epidemic include high rates of HIV drug resistance and virologic failure [21,22,23] and high rates of coinfections with HBV and TB [24,25,26,27]. Improving HIV care and clinical outcomes for PWH are key priorities of the national HIV control program, with the introduction of dolutegravir (DTG)-based ART in Sierra Leone in 2020 [23]. With NCD rates among PWH expected to continue increasing in Sierra Leone and other LMICs in the coming decades, especially given the well-known association between integrase strand inhibitor (INSTI) use and excess weight gain and metabolic complications [12], it is imperative that HIV control programs move towards NCD-aware and integrative approaches to facilitate the early detection and management of comorbidities.

The aim of this study was to assess the prevalence and associated factors of NCDs among newly diagnosed PWH prior to initiating ART at the largest HIV treatment center in Freetown, Sierra Leone. Given the high rates of coinfections in Sierra Leone, we further aimed to explore the role of HBV and TB as potential modifiers of NCD risk among PWH in this setting.

## 2. Materials and Methods

### 2.1. Study Setting, Design and Population

We analyzed sociodemographic and baseline clinical data of adults enrolled in the Sierra Leone HIV Cohort Study at the HIV Clinic at Connaught Hospital in Freetown, Sierra Leone, from March to September 2021. The HIV Clinic at Connaught Hospital is the largest HIV treatment center in Sierra Leone and is affiliated with the College of Medicine and Allied Health Sciences of the University of Sierra Leone.

The Sierra Leone HIV Cohort Study is a prospective study that was commenced in March 2021, with the primary objective of evaluating immunologic and virologic outcomes of PWH initiated on DTG-based ART in Sierra Leone. All patients who were aged ≥18 years, ART-naïve, received screening for HBV and TB and provided informed consent were eligible for inclusion into the cohort study.

### 2.2. Routine Laboratory Assessments

HIV status was determined using the rapid test by SD Bioline HIV-1/2 3.0 (Standard Diagnostics, Inc., Suwon, Korea) while HBsAg status was determined using the testing kit Citest^TM^ Diagnostics Inc. (Vancouver, BC, Canada) according to the manufacturers’ instructions. TB was defined as having at least one sputum test with a positive acid-fast bacillus (AFB) and/or positive Xpert MTB RIF test. Complete blood count (leukocytes, hemoglobin, platelets), electrolytes, serum creatinine, liver function tests (alkaline phosphatase, ALP; aspartate transaminase, AST; alanine transaminase, ALT; and gamma-glutamyl transpeptidase, GGT), bilirubin (total and direct), total protein and albumin were determined using automated analyzers by Cypress Diagnostics Inc. (Vancouver, BC, Canada). CD4 count was determined using the Alere Pima Analyzer (Abbott, Jena, Germany).

### 2.3. Assessment of Liver Disease

We used three validated non-invasive scores to screen patients for the presence of liver fibrosis and cirrhosis. The Aspartate Transaminase to Platelet Ratio (APRI) score was calculated using the formula [AST (IU/L)/AST (Upper Limit of Normal) (IU/L)]/[Platelet Count (109/L)] × 100. The following APRI thresholds were used to stage liver disease, as previously described by others [28,29,30,31]: APRI < 0.5, normal liver; APRI 0.5–1.5, significant fibrosis; and APRI > 1.5, cirrhosis. The FIB-4 score was calculated using the formula [Age (years) × AST (IU/L)]/[Platelet Count (109/L) × [ALT (IU/L)]1/2]. A FIB-4 score < 1.45 was interpreted as having normal liver, FIB-4 score of 1.45–3.25 as significant fibrosis, while FIB-4 > 3.25 as cirrhosis [28,29,30,31]. Finally, the GGT-to-platelet ratio (GPR) was calculated using the formula GGT (IU/L)/Platelet count (109/L), with threshold of GPR > 0.32 for significant fibrosis or cirrhosis as previously described by Lemoine et al. [32]. Finally, alcoholic hepatitis was defined as AST/ALT ratio > 2, as previously described [33]. We used reference values for LFTs previously reported in studies from West Africa [32,34].

### 2.4. Assessment of Renal Impairment

Glomerular filtration rate (eGFR) was estimated and staged (stages 1 to 5) using the Modification of Diet in Renal Disease (MDRD) Study equation, as follows [35]:

eGFR = 175 × (serum creatinine) − 1.154 × (age) − 0.203 × (0.742 if female) × (1.212 if black)

where GFR is expressed as mL/min/1.73 m^2^ of body surface area and serum creatinine is expressed in mg/dL. Renal impairment was defined as eGFR < 60 mL/min/1.73 m^2^ (i.e., stages 3 to 5) [36].

### 2.5. Assessment of Impaired Glucose Metabolism

We screened for impaired glucose metabolism in accordance with the American Diabetic Association (ADA)’s criteria, defined as follows: (1) prediabetes, defined as fasting blood glucose level of 5.6–6.9 mmol/L and/or HbA1c 5.7–6.4%; (2) DM, fasting blood glucose level ≥ 7.0 mmol/L, HbA1c ≥ 6.5% or being on antidiabetic medications, regardless of biomarker levels [37].

### 2.6. Other Assessments

In accordance with the American College of Cardiology/ American Heart Association (ACC/AHA) [38], hypertension was assessed using the average of two readings and/or being on treatment with antihypertensives, and was classified as follows: (1) normal: < 120/80 mmHg; (2) elevated: systolic blood pressure (SBP) > 120–129 mmHg and diastolic blood pressure (DBP) < 80 mmHg; Stage 1: SBP > 130–139 mmHg or DBP ≥ 80–89; (2) Stage 2: SBP ≥ 140 mmHg or DBP ≥ 90 mmHg. Anemia was defined as hemoglobin concentration of < 11.0 g/dL for women and < 13.0 g/dL for men aged ≥ 18 years.

### 2.7. Statistical Analyses

Statistical analyses were performed using the SPSS Version 28.0 (IBM Corp; Armonk, NY, USA). Categorical variables were reported as frequencies (percentages) and associations assessed using Pearson’s chi-square or Fisher’s exact tests. Continuous variables were presented as medians (interquartile ranges, IQR) and associations assessed using the non-parametric independent samples Mann–Whitney U-test. A logistic regression model was used to identify associations between NCDs, HBV and TB. For predicting significant liver fibrosis, we used APRI > 0.7 in accordance with Lin et al. [31] who reported a sensitivity of 77% and specificity of 72% using this threshold. Risk factors associated with NCDs were accessed in the univariate analysis. Variables that attained a *p*-value of < 0.2 in the univariate analysis were included in the multivariate regression model. Associations were reported as crude (OR) and adjusted odds ratios (aOR) with 95% confidence intervals (CI), with statistical significance set at *p* < 0.05.

### 2.8. Ethical Considerations

Ethical approval was obtained from the Sierra Leone Ethics and Scientific Review Committee (approved 10 February 2020). Written informed consent was obtained from all study participants prior to enrolment.

## 3. Results

### 3.1. Sociodemographic and Clinical Characteristics of Study Participants

Baseline data were analyzed on 275 adults aged ≥18 years newly diagnosed with HIV prior to initiating DTG-based ART (Table 1). The median age was 33 years (IQR 27–42) and the median BMI was 22 kg/m^2^ (IQR 19.8–24.3). The majority were female (56.0%, 154/275) and employed in the informal sector (69.1%, 190/275). About 23.6% (65/275) endorsed alcohol use and 20.7% (57/275) were smokers. About 15.3% (26/174) had HIV/HBV coinfection, and 8.7% (24/275) had tuberculosis. The prevalence of opportunistic conditions was low. The median CD4 cell count was 307 cells/mm^3^ (149–502), with 57.2% (119/259) meeting the criteria for late-stage HIV presentation (i.e., CD4 < 350 cells/mm^3^). Furthermore, 30.9% (80/259) had AIDS (i.e., CD4 < 200 cells/mm^3^), while 15.1% (39/259) had severe immunosuppression (i.e., CD4 < 100 cells/mm^3^).

### 3.2. Baseline Prevalence of NCDs Prior to Initiation of ART

Table 2 displays the baseline laboratory findings of study participants by coinfection status, while Table 3 provides a summary of the prevalence of individual NCDs.

### 3.3. Malnutrition, Preobesity and Obesity

Based on BMI measurements, 17.5% (48/275) were underweight (malnourished), while 22.9% (63/275) were classified as having obesity/preobesity (Table 2).

### 3.4. Hypertension and Chronic Lung Disease

About 9.8% (27/275) had elevated BP, 2.5% (7/275) had stage 1 HTN and 10.2% (28/275) had stage 2 HTN. The overall prevalence of chronic lung diseases (i.e., asthma and chronic obstructive lung disease) was low, at 1.8% (5/275) (Table 2).

### 3.5. Anemia and Thrombocytopenia

Overall, 73.5% (125/170) had anemia, while a relatively smaller proportion (9.5%, 16/169) had thrombocytopenia. There were no differences in median values based on coinfection status (Table 2).

### 3.6. Derangements in Liver Function Tests and Alcoholic Liver Disease

A large proportion of patients had deranged LFTs at baseline (Table 2). Overall, 32.3% (52/161) had elevated ALP, while 59.6% (96/161), 21.7% (35/161) and 55.9% (71/161) had elevated AST, ALT and GGT, respectively. Similarly, many participants had elevated total bilirubin (43.5%, 70/161) and direct bilirubin (55.9%, 90/161), while 10.5% (16/153) had hypoalbuminemia. There was no difference in LFT levels based on coinfection status, except for ALT (*p* < 0.016) and total bilirubin (*p* = 0.030). About 23.6% (65/275) reported regular alcohol use, while 38.5% (62/161) had AST/ALT > 2.0, suggesting a higher prevalence of alcoholic liver disease than was self-reported.

### 3.7. Liver Fibrosis and Cirrhosis

The median APRI score was 0.4 (IQR 0.2–0.5) (Table 2). The majority (71.1%, 113/159) had mild to significant fibrosis, while 1.3% (4/159) had cirrhosis. Across groups, HIV/HBV coinfected individuals tended to have a higher prevalence of liver fibrosis (34.8% vs. 29.0%) and cirrhosis (4.3% vs. 1.3%) without achieving statistical significance (*p* = 0.421). Similar results were observed for the overall prevalence of fibrosis using the FIB-4 score (23.9%, 38/159) and the GPR (25.8%, 32/124).

### 3.8. Kidney Disease and Proteinuria

The median serum creatinine and eGFR were 0.9 mg/L and 99 mL/min/1.73 m^2^, respectively, with no statistically significant differences observed across groups based on coinfection status (Table 2). About 12.6% (24/171) were classified as having renal impairment, with 7.6% (13/171) having end-stage renal disease (ESRD). Additionally, 16.7% (27/162) had proteinuria (urine dipstick ≥ +1).

### 3.9. Prediabetes and Diabetes Mellitus

The median fasting blood glucose was 4.7 mmol/L. The prevalence of DM was 5.1% (7/137) and the prevalence of prediabetes was 20.4% (28/137). Individuals with TB had significantly higher rates of prediabetes (77.8% vs. 20.4%, *p* < 0.001) (Table 2).

### 3.10. Predictors of Liver Fibrosis, Renal Impairment and Impaired Glucose Metabolism

Table 4 displays the results of univariate and multivariate regression analysis of factors associated with NCDs. In logistic regression analysis, liver fibrosis/cirrhosis was independently associated with HBV infection (aOR 8.80, 95% [2.45–31.45]; *p* < 0.001) and DM/prediabetes (aOR 9.89, 95% CI [1.14–85.67]; *p* = 0.037). DM/prediabetes was predicted by age > 35 years (aOR 2.43, 95% CI [1.01–5.84]; *p* = 0.047) and TB infection (aOR 7.34, 95% CI [1.87–28.74]; *p* = 0.004]. Lastly, renal impairment was independently associated with proteinuria (aOR 9.37, 95% CI [2.01–43.78]; *p* = 0.004) and SBP > 140 mmHg (aOR 6.00, 95% CI [1.01–35.39]; *p* = 0.049). The full univariate and multivariate analysis for associations between HBV, TB and NCDs are presented in the Supplementary Materials (Tables S1–S3).

## 4. Discussion

In this study, we estimated the burden and associated risk factors of common NCDs in a cross-section of newly diagnosed PWH prior to the initiation of ART in Freetown, Sierra Leone. The primary aim of the study was to identify opportunities for early intervention in the management of comorbidities, in a bid to foster greater integration of HIV and NCD care. Despite being a relatively young cohort (median age 33 years), the prevalence of NCDs was substantial and bimodally distributed, with about 1 in every 4 PWH screened clustered around liver disease (fibrosis/cirrhosis), impaired glucose intolerance (DM/prediabetes) and obesity/preobesity, while about 1 in 8 had renal impairment or HTN. The prevalence of chronic lung disease was low, affecting only 1.8%. Overall, 41.5% had at least one NCD, while 17.5% were multimorbid (≥2 NCDs). Previous studies from SSA have revealed similarly high rates of NCDs among PWH. A recent study from Uganda by Kansiime et al. [39] found that 20.3% of PWH initiating ART had at least one NCD. Similarly, Ekrikpo et al. [40] recently reported an NCD prevalence rate of about 25% among ART-naïve PWH in Nigeria.

In terms of individual NCDs, liver morbidity was the most significant baseline finding in our cohort. This was reflected in the high proportion of patients showing evidence of hepatic necroinflammatory activity, with 21.7% to 59.6% having elevated AST, ALT and GGT in the setting of a high prevalence of HIV/HBV coinfection (i.e., 15.3%). Similarly, the prevalence of liver fibrosis/cirrhosis was high (range, 23.9% to 30.3%) and was strongly predicted by HIV/HBV coinfection, which increased the risk of liver fibrosis almost 9-fold. As previously discussed, HBV is hyperendemic in Sierra Leone, with studies suggesting a 1.5- to 2-fold higher risk of HBV infection among PWH compared with the general population [24,25].

The high prevalence of HIV/HBV coinfection and liver morbidity among our patients has two major implications. Firstly, the elevated LFTs (i.e., ALT, AST and GGT) at baseline present challenges for the selection of regimens for the treatment of HIV, HBV and TB, given the high risk of idiosyncratic drug-related hepatoxicities commonly encountered with regimens used in the treatment of all three infections [41,42]. Secondly, given the lack of advanced diagnostic facilities in Sierra Leone for the assessment of liver disease such as liver biopsy and transient elastography, major investments are urgently needed in this area to facilitate early diagnosis and linkage to care.

About 5.1% of our cohort had DM, while 20.4% had prediabetes. DM affects 422 million people globally and is responsible for 1.5 million deaths annually [43]. The incidence of DM and related conditions such as obesity, the metabolic syndrome and CVD have been rising in the last three decades, with the impact most dramatically felt in LMICs [43]. Consequently, many LMICs have been prioritizing early screening for prediabetes, which offers opportunities for halting progression to DM through risk reduction interventions such as dietary modifications and increased physical activity. However, many LMICs lack data to help inform policy. To date, only three studies have addressed the prevalence of DM in the general population in Sierra Leone, which have ranged from 2.4% to 7.0% [44,45,46]. To the best of our knowledge, our study is the first to document the prevalence of DM among PWH in the country.

Considerable controversy exists around the association between DM/prediabetes and HIV, with some studies reporting HIV infection as an independent risk factor for the development of DM/prediabetes [47,48], while other studies have failed to demonstrate any differential effect [49,50]. To complicate the picture further, HIV-induced inflammation and ART-related toxicities (e.g., lipodystrophy seen with protease inhibitor use and INSTI-related weight gain with its resultant cardiometabolic complications) have been implicated in promoting hyperglycemia and increased insulin resistance [10,51]. Additionally, many of the risk factors traditionally associated with the development of DM/prediabetes in the general population (e.g., older age, increased BMI, genetics) also tend to be highly prevalent among PWH. However, in our study, we did not detect statistically significant associations among these factors, despite the well-described link among increased BMI, DM/prediabetes and CVD [52,53]. Studies have shown that waist circumference alone or combined with BMI is a better predictor of cardiovascular and all-cause morbidity and mortality risk than BMI alone, especially at a BMI < 35 kg/m^2^ [54,55], which could partially explain our findings.

We also found that patients with DM/prediabetes were at 10 times higher risk of having liver fibrosis/cirrhosis, compared with their normoglycemic counterparts. Our findings align with several studies that have correlated poor glycemic control with liver disease, especially in cirrhotic patients [56,57]. The mechanism underlying this phenomenon appears to be tied to the central role of the liver in regulating glucose metabolism through glycogenesis and glycogenolysis [58]. In liver disease, glucose metabolism shifts from the liver to muscle and adipose tissue, which stimulates mitochondrial oxidative stress and drives the production of proinflammatory adipokines, e.g., leptin, tumor necrosis factors-alpha and interleukin-6 [59]. Adipokine release leads to the activation of hepatic stellate cells and the excess production and deposition of collagen and extracellular matrix, culminating in liver fibrosis [60,61].

Another important finding in our study was that PWH with TB had a 7.34-fold higher risk of DM/prediabetes compared with patients without TB. DM is a growing global health concern among people with TB, with a recent meta-analysis by Noubiap et al. [62] estimating that 15.3% of TB patients worldwide had DM. The association between TB and DM is well known; however, data are conflicting on the direction of the relationship. Diabetics are 1.5 to 3 times more likely to develop active TB [63,64]. Conversely, a meta-analysis by Menon et al. [65] showed that up to 50% of people diagnosed with TB had hyperglycemia at baseline, which remained unresolved in over 10% of cases even after 6 months of effective anti-TB treatment. Although the pathophysiologic basis of hyperglycemia/DM in TB has not been fully elucidated, a role has been postulated for adipokine-mediated alterations in the microenvironment, resulting in increased susceptibility to hyperglycemia through the release of counterregulatory stress hormones (e.g., glucagon, cortisol and growth hormone) [66,67]. Others have suggested that TB-induced impaired cell-mediated immune responses have a direct effect on pancreatic islet cells, resulting in endocrine hypofunction, including a reduction in insulin production [68]. In response to the rising rates of DM in TB-endemic regions, the World Health Organization (WHO) has proposed a collaborative framework, with the aim of achieving greater integration of DM and TB care in LMICs [69].

Renal impairment and HTN (stages 1 and 2) occurred at equal rates in our cohort, affecting about 12.6% (1 in every 8) of PWH. While a proportion of patients will see an improvement in renal function with treatment, many are likely to progress to chronic kidney disease (CKD). CKD is common in HIV infection and is estimated to affect 6.4% of PWH globally, with countries in West Africa reporting the highest prevalence (i.e., 14.6%) [70]. Proteinuria was present in 16.7% of our patients and conferred a 9.34-fold higher risk of kidney disease, suggesting HIV-associated nephropathy (HIVAN) as a major contributor to kidney disease in this setting. HIVAN has a predilection for African populations and has been linked with high-risk variants of the APOL1 gene (i.e., G1 and G2), which have been associated with several nondiabetic glomerular disorders including HIVAN [70,71]. Studies in West African populations, notably Ghana and Nigeria, have found APOL1 allele frequencies of up to 40% [72,73]. Notwithstanding this, CKD was also strongly predicted by HTN (SBP > 140 mmHg) by a factor of 6, in line with the observation that renal disease tends to be multifactorial. However, we did not detect statistically significant associations with other factors traditionally linked with kidney disease such as age, DM or HBV status. Our findings have implications for the use of tenofovir disoproxil fumarate (TDF) in the treatment of HIV and HBV, given that less nephrotoxic treatment options such as tenofovir alafenamide fumarate (TAF) and emtricitabine (FTC) are currently not available in Sierra Leone and many other LMICs.

Our study had a few limitations worthy of discussion. Firstly, the study was restricted to a single treatment center and may not be generalizable to other geographic locations in Sierra Leone. Secondly, we used non-invasive scores to assess liver disease due to the unavailability of more precise diagnostic tools such as liver biopsy and transient elastography in Sierra Leone. To improve the precision of our estimates for the prevalence of liver disease, we used three non-invasive scores that have been well-validated in West Africa [28,29,30,31,32]. Thirdly, we were unable to fully assess CVD risk, given that the lipid profile and waist circumference, which are better indicators of health risk, were not routinely measured in this relatively young cohort. Finally, we were unable to assess the impact of HIV viremia on NCDs since, at baseline, HIV viral load measurement was not routinely available prior to ART initiation. However, we do not believe that this is a major limitation. As most patients presented with late-stage HIV disease (60% with CD4 < 350 cells/mm^3^), we assumed that most would have had a high viral load at diagnosis, given the inverse relationship of CD4 count and HIV viremia in untreated HIV infection [74]. This would have likely made it difficult to discern any differential effect between NCDs and HIV viremia. Despite these limitations, our study is the first to characterize NCDs among PWH in Sierra Leone and offers important insights into the link between coexisting endemic infections to NCDs in this setting. This warrants the implementation of HIV programs emphasizing greater integrative care in LMICs, with a focus on the early detection and management of comorbidities.

## 5. Conclusions

In summary, our study found a high burden of NCDs among PWH prior to the initiation of ART in Freetown, Sierra Leone, with 1 in every 4 who were screened clustering around liver fibrosis/cirrhosis, DM/prediabetes and obesity/preobesity, while about 1 in 8 had CKD and/or HTN. The prevalence of chronic lung disease was low, affecting only 1.8%. Overall, 41.5% had at least one NCD, while 17.5% were multimorbid (≥2 NCDs). HBV coinfection increased the risk of liver disease (fibrosis/cirrhosis) 9-fold, while TB coinfection conferred a 7.3-fold increase in the risk of DM/prediabetes, which was in turn associated with a 10-fold increase in the risk of liver disease. Larger studies are needed to confirm these findings. Additionally, given the substantially high burden of NCDs among this relatively young cohort of PWH and their link to HBV and TB coinfections, this warrants the implementation of HIV programs emphasizing greater integrative care in Sierra Leone and other LMICs, with a renewed focus on the prevention, early detection and management of comorbidities.

## Figures and Tables

**Table 1:** Sociodemographic and clinical characteristics of study participants.

Variables	N (%)
**Gender**, *n* (%)	
Male	121/275 (44.0)
Female	154/275 (56.0)
**Age**, *years,n* (%)	
Median (IQR)	33 (27–42)
<25	42/275 (15.3)
25–34	110/275 (40.0)
35–44	70/275 (25.5)
45–54	42/275 (15.3)
≥55	11/275 (4.0)
**Highest education attained**, *n* (%)	
None	52/275 (18.9)
Primary	40/275 (14.5)
Secondary	128/275 (46.5)
Tertiary	55/275 (20.0)
**Employment status**, *n* (%)	
Unemployed	57/275 (20.7)
Informal	190/275 (69.1)
Formal	28/275 (10.2)
**Monthly earning**, *n* (%)	
<USD 100	217/275 (78.9)
≥USD 100	58/275 (21.1)
**Body mass index**, *kg/m^2^*, *n* (%)	
Median (IQR)	22.0 (19.8–24.3)
<18.5	48/275 (17.5)
18.5–24.9	164/275 (59.6)
25.0–29.9	43/275 (15.6)
≥30.0	20/275 (7.3)
**Lifestyle-associated risk factors**, *n* (%)	
Smoking	57/275 (20.7)
Alcohol use	65/275 (23.6)
Drug use	28/275 (10.2)
**Coinfections**, *n* (%)	
HBV	26/174 (15.3)
Tuberculosis	24/275 (8.7)
**CD4 count**, *cells/mm^3^*, *n* (%)	
Median (IQR)	307 (149–502)
0–99	39/259 (15.1)
100–199	41/259 (15.8)
200–349	68/259 (26.3)
≥350	111/259 (42.9)

Abbreviations: HBV, hepatitis B virus; IQR, interquartile range; USD, United States dollars.

**Table 2:** Baseline laboratory parameters of study participants.

Laboratory Parameters	All	HIV Only	HIV/HBV	HIV/TB	*p*-Value
N	275	225	26	24	
**Leukocytes**, *×10^9^/L*, *n* (%)					
Median (IQR)	5.1 (4.0–6.4)	5.1 (4.1–6.4)	5.3 (4.4–6.1)	4.8 (3.0–6.5)	0.745
**Hemoglobin**, *g/dL*, *n* (%)					
Median (IQR)	11.2 (9.5–12.4)	11.1 (9.5–12.7)	11.7 (10.9–12.3)	10.1 (8.4–11.9)	0.204
Anemia	125/170 (73.5)	94/131 (71.8)	17/23 (73.9)	12/12 (85.7)	0.532
**Platelets**, *×10^9^/L*, *n* (%)					
Median (IQR)	268 (209–344)	272 (198–347)	253 (225–306)	254 (223–410)	0.821
Thrombocytopenia	16/169 (9.5)	13/131 (9.9)	1/23 (4.3)	1/13 (7.7)	0.880
**ALP**, *U/L*, *n* (%)					
Median (IQR)	96 (79–143)	95 (77–135)	96 (78–168)	104 (87–135)	0.467
Elevated	52/161 (32.3)	36/124 (29.0)	10/23 (43.5)	4/12 (33.3)	0.379
**AST**, *U/L*, *n* (%)					
Median (IQR)	33 (23–47)	32 (22–44)	34 (25–59)	44 (30–65)	0.276
Elevated	96/161 (59.6)	73/124 (58.9)	16/23 (69.6)	5/12 (41.7)	0.279
**ALT**, *U/L*, *n* (%)					
Median (IQR)	18 (13–27)	18 (13–27)	25 (18–47)	17 (13–20)	0.016
Elevated	35/161 (21.7)	25/124 (20.2)	10/23 (43.5)	-	0.007
**GGT**, *U/L*, *n* (%)					
Median (IQR)	38 (27–59)	37 (28–53)	40 (23–112)	44 (30–65)	0.707
Elevated	71/127 (55.9)	54/99 (54.5)	9/18 (50.0)	7/9 (77.8)	0.356
**AST/ALT**, *n* (%)					
Median (IQR)	1.8 (1.2–2.5)	1.8 (1.2–2.5)	1.3 (1.0–1.9)	2.0 (1.6–2.8)	0.064
>2.0	62/161 (38.5)	49/124 (39.5)	5/23 (21.7)	6/12 (50.0)	0.167
**Total bilirubin**, *mg/dL*, *n* (%)					
Median (IQR)	1.1 (0.8–1.8)	1.1 (0.7–1.8)	1.4 (1.0–2.2)	0.9 (0.6–1.0)	0.030
Elevated	70/161 (43.5)	55/124 (44.4)	13/23 (56.5)	2/12 (16.7)	0.078
**Direct bilirubin**, *mg/dL*, *n* (%)					
Median (IQR)	0.4 (0.2–1.0)	0.4 (0.2–1.0)	0.5 (0.3–1.4)	0.3 (0.2–0.7)	0.233
Elevated	90/161 (55.9)	68/124 (54.8)	16/23 (69.6)	6/12 (50.0)	0.378
**Total protein**, *mg/dL*, *n* (%)					
Median (IQR)	7.1 (6.8–7.7)	7.1 (6.8–7.7)	7.0 (6.7–7.7)	7.3 (6.9–8.4)	0.712
**Albumin**, *mg/dL*, *n* (%)					
Median (IQR)	3.8 (3.7–3.9)	3.8 (3.7–3.9)	3.8 (3.6–4.0)	3.8 (3.6–4.2)	0.644
Hypoalbuminemia	16/153 (10.5)	12/121 (9.9)	3/20 (15.0)	1/11 (9.1)	0.781
**Fasting blood glucose**, *mmol/L*, *n* (%)					
Median (IQR)	4.7 (4.0–5.5)	4.6 (4.0–5.4)	4.8 (4.0–5.5)	6.1 (5.0–6.5)	0.063
<5.5 (normal)	102/137 (74.5)	82/106 (77.4)	16/20 (80.0)	2/9 (22.2)	<0.001
5.6–6.9 (prediabetes)	28/137 (20.4)	18/106 (17.0)	3/20 (15.0)	7/9 (77.8)	
≥7.0 (diabetes)	7/137 (5.1)	6/106 (5.7)	1/20 (5.0)	-	
**Serum creatinine**, *mg/dL*, *n* (%)					
Median (IQR)	0.9 (0.8–1.2)	0.9 (0.8–1.2)	1.0 (0.8–1.2)	1.0 (0.7–1.3)	0.884
**eGFR**, *mL/min/1.73 m^2^*, *n* (%)					
Median (IQR)	99 (77–119)	97 (76–118)	109 (78–121)	100 (87–128)	0.569
≥90 (Stage 1)	106/171 (62.0)	83/134 (61.9)	14/23 (60.9)	8/12 (66.7)	0.724
60–89 (Stage 2)	41/171 (24.0)	30/134 (22.4)	8/23 (34.8)	2/12 (16.7)	
45–59 (Stage 3a)	6/171 (2.5)	4/134 (3.0)	1/23 (4.3)	1/12 (8.3)	
30–44 (Stage 3b)	3/171 (1.8)	3/134 (2.2)	-	-	
29–15 (Stage 4)	2/171 (0.7)	2/134 (1.5)	-	-	
<15 (Stage 5)	13/171 (7.6)	12/134 (9.0)	-	1/12 (8.3)	
**Proteinuria**, *n* (%)	27/162 (16.7)	20/126 (15.9)	4/22 (18.2)	3/12 (25.0)	0.763
**APRI**, *n* (%)					
Median (IQR)	0.4 (0.2–0.5)	0.3 (0.2–0.5)	0.4 (0.3–0.9)	0.4 (0.2–0.6)	0.296
<0.5 (normal liver)	113/159 (71.1)	90/122 (73.8)	14/23 (60.9)	9/12 (75.0)	0.421
0.5–1.5 (fibrosis)	46/159 (29.0)	31/122 (25.4)	8/23 (34.8)	3/12 (25.0)	
>1.5 (cirrhosis)	2/159 (1.3)	1/22 (0.8)	1/23 (4.3)	-	
**FIB-4 score**, *n* (%)					
Median (IQR)	1.0 (0.6–1.4)	1.0 (0.6–1.4)	0.8 (0.6–1.4)	0.9 (0.6–1.3)	0.930
<1.45 (normal liver)	121/159 (76.1)	96/122 (78.7)	15/23 (65.2)	10/12 (83.3)	0.481
1.45–3.25 (fibrosis)	38/159 (23.9)	23/122 (18.9)	8/23 (34.8)	2/12 (16.7)	
>3.25 (cirrhosis)	4/159 (1.5)	3/122 (2.5)	-	-	
**GPR**, *n* (%)					
Median (IQR)	0.45 (0.29–0.72)	0.41 (0.29–0.65)	0.49 (0.25–1.22)	0.51 (0.45–0.69)	0.430
<0.32 (normal liver)	92/124 (74.2)	72/97 (74.2)	12/18 (66.7)	7/8 (87.5)	0.532
≥0.32 (fibrosis)	32/124 (25.8)	25/97 (25.8)	6/18 (33.3)	1/8 (12.5)	

Abbreviations: ALP, alkaline phosphatase; ALT, alanine transaminase; APRI, aspartate transaminase to platelet index; AST, aspartate transaminase; AST/ALT, aspartate transaminase to alanine transaminase ratio; BMI, body mass index; eGFR, estimated glomerular filtration rate; FIB-4, fibrosis-4 score; GGT, gamma-glutamyl transferase; GPR, gamma-glutamyl transferase to platelet ratio; HBV, hepatitis B virus, HIV, human immunodeficiency virus; IQR, interquartile range.

**Table 3:** Prevalence of NCDs by type.

Type of NCD	N (%)
≥1 NCD	113/275 (41.5)
≥2 NCDs	48/275 (17.5)
Liver fibrosis/cirrhosis (APRI > 0.5)	48/159 (30.3)
Diabetes/prediabetes (FBG > 5.5 mmol/L)	35/137 (25.5)
Preobesity/obesity (BMI ≥ 25 kg/m^2^)	63/275 (22.9)
Underweight/malnutrition (BMI < 18.5 kg/m^2^)	48/275 (17.5)
Renal impairment (eGFR < 60 mL/min/1.72 m^2^)	24/171 (12.6)
Hypertension *	
Elevated blood pressure	27/275 (9.8)
Stage 1	7/275 (2.5)
Stage 2	28/275 (10.2)
Chronic lung disease	5/275 (1.8)

Abbreviations: APRI, aspartate transaminase to platelet index; BMI, body mass index; eGFR, estimated glomerular filtration rate; FBG, fasting blood glucose; * hypertension classification was based on the AHA 2019 guidelines [38].

**Table 4:** Factors associated with NCDs.

Type of NCD	Risk Factors	n (%)		Univariate Analysis		Multivariate Analysis	
		Yes	No	Crude Odds Ratio (95% CI)	*p*-Value	Adjusted Odds Ratio (95% CI)	*p*-Value
Liver fibrosis (APRI > 0.7)	**HBV**						
	Yes	10 (43.5)	15 (11.4)	6.00 (2.24–16.05)	<0.001	8.80 (2.46–31.45)	<0.001
	No	13 (56.5)	117 (88.6)	Ref		Ref	
	**Diabetes/prediabetes**						
	Yes	20 (95.2)	82 (71.3)	8.05 (1.04–62.44)	0.020	9.89 (1.14–85.67)	0.037
	No	1 (4.8)	33 (28.7)	Ref		Ref	
Diabetes/prediabetes (FBG > 5.5 mmol/L)	**Age > 35 years**						
	Yes	22 (62.9)	44 (43.1)	2.23 (1.01–4.91)	0.044	2.43 (1.01–5.84)	0.047
	No	13 (37.1)	58 (56.9)	Ref		Ref	
	**Tuberculosis**						
	Yes	7 (20.0)	4 (3.9)	6.13 (1.67–22.44)	0.003	7.34 (1.87–28.74)	0.004
	No	28 (80.0)	98 (96.1)	Ref		Ref	
Renal impairment (eGFR < 60 mL/min/1.72m^2^)	**Age > 35 years**						
	Yes	16 (66.7)	61 (41.5)	2.82 (1.14–7.00)	0.022	5.90 (1.12–31.10)	0.036
	No	8 (33.3)	86 (58.5)	Ref		Ref	
	**SBP > 140 mmHg**						
	Yes	6 (25.0)	12 (8.2)	3.72 (1.24–11.11)	0.013	5.22 (1.17–23.33)	0.031
	No	18 (75.0)	134 (91.8)	Ref		Ref	

Abbreviations: APRI, aspartate transaminase to platelet index; eGFR, estimated glomerular filtration rate; FBG, fasting blood glucose; Ref, reference category; SBP, systolic blood pressure.

## Data Availability

The data presented in this study are available on request from the corresponding author.

## Supplementary Materials

The following supporting information can be downloaded at: https://www.mdpi.com/article/10.3390/jcm11123466/s1, Table S1: Factors associated with liver fibrosis. Table S2: Factors associated with renal impairment. Table S3: Factors associated with impaired glucose metabolism.
