# Supplementary material for: Hepatitis B Virus and Tuberculosis Are Associated with Increased Noncommunicable Disease Risk among Treatment-Naïve People with HIV: Opportunities for Prevention, Early Detection and Management of Comorbidities in Sierra Leone
Source: J Clin Med. 2022 Jun 16;11(12):3466. doi: 10.3390/jcm11123466 (PMC9225550; doi:10.3390/jcm11123466)
Supplement: Supplementary file 1 [file jcm-11-03466-s001.zip › jcm-1682621-supplementary.pdf]

Supplementary Table S1. Factors associated with liver fibrosis

Risk factors	APRI > 0.7 n (%)		Univariate analysis		Multivariate analysis	
	Yes	No	OR (95% CI)	p-Value	AOR (95% CI)	p-Value
**Gender**						
Male	15 (62.5)	53 (39.3)	2.58 (1.05-6.32)	**0.034**	2.88 (0.98-8.46)	0.054
Female	9 (37.5)	82 (60.7)	Ref		Ref	
**Age**, *years*						
< 45	20 (83.3)	107 (79.3)	Ref	0.646		
≥ 45	4 (16.7)	28 (20.7)	0.76 (0.24-2.42)			
**BMI**, *kg/m²*						
<30	23 (95.8)	120 (88.9)	Ref	0.297		
≥ 30	1 (4.2)	15 (11.1)	0.35 (0.04-2.76)			
**Education**						
Secondary or less	16 (66.7)	111 (82.2)	Ref	0.080	Ref	0.264
Tertiary	8 (33.3)	24 (17.8)	2.31 (0.89-6.02)		2.04 (0.59-7.10)	
**Occupation**						
Unemployed	6 (25.0)	28 (20.7)	1.27 (0.46-3.51)	0.639		
Employed	18 (75.0)	107 (79.3)	Ref			
**Socioeconomic status**						
Low	19 (79.2)	109 (80.7)	0.91 (0.31-2.65)	0.858		
Middle/High	5 (20.8)	26 (19.3)	Ref			
**Alcohol use**						
Yes	7 (29.2)	31 (23.0)	1.38 (0.52-3.63)	0.511		
No	17 (70.8)	104 (77.0)	Ref			
**Smoking**						
Yes	4 (16.7)	33 (24.4)	0.62 (0.20-1.94)	0.406		
No	20 (83.3)	102 (75.6)	Ref			
**Drug use**						
Yes	2 (8.3)	16 (11.9)	0.68 (0.15-3.15)	0.616		
No	22 (91.7)	119 (88.1)	Ref			
**Hypertension**						
Yes	8 (33.3)	27 (20.0)	2.00 (0.78-5.16)	0.146	3.51 (0.97-12.66)	0.056
No	16 (66.7)	108 (80.0)	Ref		Ref	
**Tuberculosis**						
Yes	3 (12.5)	11 (8.1)	1.61 (0.41-6.26)	0.488		
No	21 (87.5)	124 (91.9)	Ref			
**HBV**						
Yes	10 (43.5)	15 (11.4)	6.00 (2.24-16.05)	**<0.001**	8.80 (2.46-31.45)	**<0.001**
No	13 (56.5)	117 (88.6)	Ref		Ref	
**Prediabetes/DM**						
Yes	20 (95.2)	82 (71.3)	8.05 (1.04-62.44)	**0.020**	9.89 (1.14-85.67)	**0.037**
No	1 (4.8)	33 (28.7)	Ref		Ref	
**eGFR < 60 mL/min/1.73m²**						
Yes	2 (8.3)	15 (11.1)	0.73 (0.16-3.40)	0.685		
No	22 (91.7)	120 (88.9)	Ref			
**Albumin,** mg/dL						
Normal	19 (86.4)	115 (89.8)	Ref	0.888		
Low	3 (13.6)	13 (10.2)	1.40 (0.36-5.38)			
**Direct bilirubin,** mg/dL						
Normal	12 (50.0)	59 (43.7)	Ref	0.568		
Elevated	12(50.0)	76 (56.3)	0.78 (0.33-1.85)			
**Total bilirubin,** mg/dL						
Normal	9 (37.5)	59 (43.7)	Ref	0.571		

Elevated	15 (62.5)	76 (56.3)	1.29 (0.53-3.16)	
**CD count,** cells/mm³				
<350	14 (58.3)	73 (54.1)	1.19 (0.49-2.287)	0.699
≥350	10 (41.7)	62 (45.9)	Ref	

Supplementary Table S2. Factors associated with renal impairment

Risk factors	eGFR < 60 ml/min/1.73m²		Univariate analysis		Multivariate analysis	
	Yes	No	OR (95% CI)	p-Value	AOR (95% CI)	p-Value
**Gender**						
Male	8 (33.3)	65 (44.2)	0.63 (0.25-1.157)	0.318		
Female	16 (66.7)	82 (55.8)	Ref			
**Age > 45 years**						
Yes	9 (37.5)	25 (17.0)	2.93 (1.15-7.43)	**0.020**	3.23 (0.73-14.30)	0.122
No	15 (62.5)	122 (83.0)	Ref		Ref	
**BMI**, *kg/m²*						
<30	22 (91.7)	133 (90.5)	Ref	0.853		
≥ 30	2 (8.3)	14 (9.5)	0.86 (0.18-4.07)			
**Education**						
Secondary or less	16 (66.7)	121 (82.3)	0.43 (0.17-1.11)	0.**075**	0.49 (0.09-2.52)	0.389
Tertiary	8 (33.3)	26 (17.7)	Ref		Ref	
**Occupation**						
Unemployed	3 (12.5)	33 (22.4)	0.49 (0.14-1.76)	0.268		
Employed	21 (87.5)	114 (77.6)	Ref			
**Socioeconomic status**						
Low	20 (83.3)	119 (81.0)	1.18 (0.37-3.72)	0.782		
Middle/High	4 (16.7)	28 (19.0)	Ref			
**Alcohol use**						
Yes	5 (20.8)	35 (23.8)	0.84 (0.29-2.42)	1.000		
No	19 (79.2)	112 (76.2)	Ref			
**Smoking**						
Yes	5 (20.8)	34 (23.1)	0.88 (0.30-2.52)	1.000		
No	119 (79.2)	113 (76.9)	Ref			
**SBP > 140 mmHg**						
Yes	6 (25.0)	12 (8.2)	3.72 (1.24-11.11)	**0.013**	6.00 (1.01-35.39)	**0.049**
No	18 (75.0)	134 (91.8)	Ref		Ref	
**Tuberculosis**						
Yes	2 (8.3)	12 (8.2)	1.02 (0.21-4.88)	0.978		
No	22 (91.7)	135 (91.8)	Ref			
**Anemia**						
Yes	20 (90.9)	102 (70.3)	4.22 (0.94-18.83)	**0.043**	4.78 (0.44-52.13)	0.200
No	2 (9.1)	43 (29.7)	Ref		Ref	
**Thrombocytopenia**						
Yes	4 (18.2)	12 (8.3)	2.46 (0.72-8.46)	0.141	2.83 (0.39-20.32)	0.302
No	18 (81.8)	133 (91.7)	Ref		Ref	
**HBV**						
Yes	1 (5.6)	24 (17.0)	0.29 (0.04-2.26)	0.208		
No	17 (94.4))	117 (83.0)	Ref			
**Prediabetes/DM**						
Yes	6 (40.0)	29 (23.8)	2.14 (0.70-6.51)	0.174	2.07 (0.51-8.41)	0.307
No	9 (60.0)	93 (76.2)	Ref		Ref	
**Albumin,** mg/dL						

Normal	12 (80.0)	124 (90.5)	Ref	0.208		
Low	3 (20.0)	13 (9.5)	2.39 (0.59-9.52)			
**Proteinuria**						
Yes	11 (55.0)	15 (10.7)	10.19 (3.63-28.56)	**<0.001**	9.37 (2.01-43.78)	**0.004**
No	9 (45.0)	125 (89.3)	Ref		Ref	
**CD4 < 100 cells/mm³**						
Yes	6 (26.1)	15 (10.2)	3.12 (1.06-9.08)	**0.031**	2.37 (0.37-15.33)	0.365
No	17 (73.9)	132 (89.8)	Ref		Ref	

Abbreviations: APRI, aspartate transaminase to platelet index; BMI, body mass index; eGFR, estimated glomerular filtration rate; ESRD, end stage liver disease; HBV, hepatitis B virus; Ref, reference category

Supplementary Table S3. Factors associated with impaired glucose metabolism

**Risk factors**	**Prediabetes/diabetes n (%)**		**Univariate analysis**		**Multivariate analysis**	
	**Yes**	**No**	**OR (95% CI)**	**p-Value**	**AOR (95% CI)**	**p-Value**
**Gender**						
Male	15 (42.9)	41 (40.2)	1.12 (0.51-2.43)	0.782		
Female	20 (57.1)	61 (59.8)	Ref			
**Age > 35 years**						
Yes	22 (62.9)	44 (43.1)	2.23 (1.01-4.91)	**0.044**	2.43 (1.01-5.84)	**0.047**
No	13 (37.1)	58 (56.9)			Ref	
**BMI ≥ 30 kg/m²**						
Yes	5 (14.3)	93 (91.2)	1.72 (0.54-5.52)	0.348		
No	30 (85.7)	9 (8.8)	Ref			
**Educational level**						
Secondary or lower	7 (20.0)	80 (78.4)	0.91 (0.35-2.36)	0.845		
Tertiary	28 (80.0)	22 (21.6)	Ref			
**Employment status**						
Unemployed	6 (17.1)	23 (22.5)	0.71 (0.26-1.92)	0.499		
Employed	29 (82.9)	79 (77.5)	Ref			
**Socioeconomic status**						
Low	28 (80.0)	85 (83.3)	0.80 (0.30-2.13)	0.654		
Middle/high	7 (20.0)	17 (16.7)	Ref			
**Alcohol**						
Yes	9 (25.7)	21 (20.6)	1.34 (0.54-3.28)	0.527		
No	26 (74.3)	81 (79.4)	Ref			
**Smoking**						
Yes	8 (22.9)	21 (20.6)	1.14 (0.45-2.88)	0.777		
No	27 (77.1)	81 (79.4)	Ref			
**Drug use**						
Yes	5 (14.3)	8 (7.8)	1.96 (0.60-6.44)	0.316		
No	30 (85.7)	94 (92.2)	Ref			
**HBV**						
Yes	4 (11.4)	18 (17.8)	0.60 (0.19-1.90)	0.438		
No	31 (88.6)	83 (82.2)	Ref			
**Tuberculosis**						
Yes	7 (20.0)	4 (3.9)	6.13 (1.67-22.44)	**0.003**	7.34 (1.87-28.74)	**0.004**
No	28 (80.0)	98 (96.1)	Ref		Ref	
**SBP > 140 mmHg**						
Yes	3 (8.6)	10 (9.8)	0.86 (0.22-3.33)	1.000		
No	32 (91.4)	92 (90.2)	Ref			
**eGFR < 60 mL/min/1.73m²**						

Yes	6 (17.1)	9 (8.8)	2.14 (0.70-6.51)	**0.174**	1.165 (0.50-5.48)	0.413
No	29 (82.9)	93 (91.2)	Ref		Ref	
**APRI > 0.5**						
Yes	7 (20.6)	32 (31.4)	0.57 (0.22-1.44)	0.229		
No	27 (79.4)	70 (68.6)	Ref			
**CD4 < 200 cells/mm^3**						
Yes	12 (34.3)	22 (21.6)	1.90 (0.82-4.41)	0.**133**	1.80 (0.73-4.34)	0.206
No	23 (65.7)	80 (78.4)	Ref		Ref	
